# Supplementary material for: Identification and functional analysis of non-coding regulatory small RNA FenSr3 in Bacillus amyloliquefaciens LPB-18
Source: PeerJ. 2023 May 15;11:e15236. doi: 10.7717/peerj.15236 (PMC10194069; doi:10.7717/peerj.15236)
Supplement: Supplemental Information 4 [file peerj-11-15236-s004.zip › KO/CK-vs-T1_map/map04720.html]

KEGG PATHWAY: Long-term potentiation - Reference pathway


|  |  |
| --- | --- |
| **Long-term potentiation - Reference pathway** |  |

[
Pathway menu
| Organism menu
| Pathway entry
| Show description
| User data mapping
]

Hippocampal long-term potentiation (LTP), a long-lasting increase in synaptic efficacy, is the molecular basis for learning and memory. Tetanic stimulation of afferents in the CA1 region of the hippocampus induces glutamate release and activation of glutamate receptors in dendritic spines. A large increase in [Ca2+]i resulting from influx through NMDA receptors leads to constitutive activation of CaM kinase II (CaM KII) . Constitutively active CaM kinase II phosphorylates AMPA receptors, resulting in potentiation of the ionic conductance of AMPA receptors. Early-phase LTP (E-LTP) expression is due, in part, to this phosphorylation of the AMPA receptor. It is hypothesized that postsynaptic Ca2+ increases generated through NMDA receptors activate several signal transduction pathways including the Erk/MAP kinase and cAMP regulatory pathways. The convergence of these pathways at the level of the CREB/CRE transcriptional pathway may increase expression of a family of genes required for late-phase LTP (L-LTP).

|  |  |  |
| --- | --- | --- |
| Reference pathway | 184% 150% 122% 100% 82% 67% 55% | 图片下载 |
